# Sex and Depot Specific Adipocyte Proteome Profiling In Vivo via Intracellular Proximity Labeling

**DOI:** 10.1002/cph4.70007

**Published:** 2025-04-03

**Authors:** Taylor L. Simonian, Amanda S. Meyer, Jinjin Guo, Jihui Sha, James A. Wohlschlegel, Ilia A. Droujinine, Norbert Perrimon, Andrew P. McMahon

**Affiliations:** ^1^ Department of Stem Cell Biology and Regenerative Medicine, Eli and Edythe Broad Center for Regenerative Medicine and Stem Cell Research, Keck School of Medicine University of Southern California Los Angeles California USA; ^2^ Department of Biological Chemistry, David Geffen School of Medicine University of California Los Angeles California USA; ^3^ Department of Molecular Medicine Scripps Research Institute La Jolla California USA; ^4^ Department of Genetics, Blavatnik Institute Harvard Medical School Cambridge Massachusetts USA; ^5^ Howard Hughes Medical Institute Chevy Chase Maryland USA

**Keywords:** adipocytes, adipose tissue, mouse model, proteomics, proximity labeling

## Abstract

Adipose tissue has varying distributions and metabolic properties between the sexes. Inherent sex‐specific differences in adipocytes may heighten the risk of metabolic disease in males. Analysis of the adipocyte proteome can potentially provide important insight. To enable cell‐type specific proteomic profiling in vivo, we genetically engineered a mouse line for cell‐type specific production of a promiscuous biotin ligase (BirA*G3) facilitating the rapid isolation of biotinylated cell‐type specific proteomes. Adipocyte‐specific activation of cytoplasmic BirA*G3 led to robust biotinylation of adipocyte proteins across all major fat depots. Comparison of brown adipose tissue (BAT) and subcutaneous white adipose tissue (SAT) proteomes identified 229 brown adipose‐enriched and 35 white adipose‐enriched proteins. Regional comparison of white fat depots revealed additional differences across depots. Comparison of male and female depots identified sexually dimorphic adipose proteins: AHNAK predominating in the male and ACOT2 in the female. These findings validate the genetic model and highlight insights to be gained through targeted profiling of adipocytes. The genetic tool adds to existing approaches for in vivo proximity profiling of cell‐type specific proteome programs.

## Graphical abstract

We report a mouse model in which a promiscuous biotin ligase, BirA*G3, allows for facile proximity labeling and proteomic profiling of potentially any cell type. This method adds to a growing set of technologies bringing proteome‐scale analysis to physiological mammalian systems.

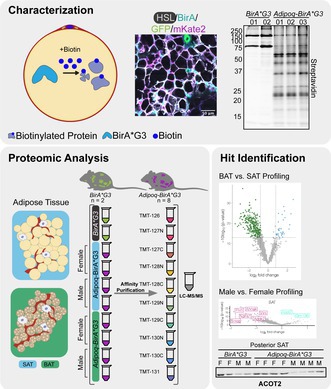

## 1 Introduction

Adipose tissue plays critical roles in energy balance and homeostasis, is highly plastic, differing between the sexes, and adipocyte function is perturbed in several major diseased states (Fuente‐Martín et al. 2013; Fuster et al. 2016; Coelho et al. 2013; Sonne et al. 2017). Adipocytes secrete endocrine adipokines that act on specific target tissues to regulate metabolism and energy expenditure (Coelho et al. 2013; Sonne et al. 2017). The abundance, size, and distribution of adipocytes in different fat depots change in response to aging, environmental conditions, and vary between the sexes (Fuster et al. 2016).

The fat depots are comprised of brown (BAT) or white (WAT) adipose tissue. BAT is involved in energy expenditure and thermogenesis primarily through the activation of uncoupling protein 1 (UCP1) (Sonne et al. 2017). WAT is critical for energy storage within subcutaneous (SAT) and visceral (VAT) WAT depots. SAT is located directly beneath the skin, whereas VAT surrounds intra‐abdominal organs, where excessive VAT is a strong predictor of the development of metabolic diseases (Sonne et al. 2017; Villarroya et al. 2017; Coolbaugh et al. 2019; Fabbiano et al. 2016). Females have a higher body fat percentage throughout life compared to males and have increased adiposity in the subcutaneous abdominal and gluteal regions (Fasshauer and Blüher 2015). They also have higher amounts of brown and beige adipose tissue. In obesity, adipose tissue undergoes rapid expansion to compensate for the excessive caloric intake (Sonne et al. 2017; Fabbiano et al. 2016; Fasshauer and Blüher 2015). This leads to numerous pathologies within adipose tissue, including hypoxia, inflammation, and dyslipidemia (Weisberg et al. 2003; Chouchani and Kajimura 2019). As a result, adipose tissue fails to function normally. Depot‐specific obesity changes have also been documented, but the full extent of depot‐specific pathological contribution remains unknown (Sonne et al. 2017; Chouchani and Kajimura 2019). Interestingly, research has suggested that males may be at a higher risk for developing metabolic disease and obesity‐related disorders (Benites‐Zapata et al. 2019; Schorr et al. 2018; Regitz‐Zagrosek et al. 2006; Eguchi et al. 2011). Though adipocytes have been well‐studied, structural features of adipocytes and the mosaic cell composition of adipose tissue make specific proteomic profiling of functioning adipocytes in vivo a challenge.

Adipocyte profiling requires tissue digestion and dissociation followed by fractionation, resulting in an extended processing time, which may alter the state of the proteome. Adipocytes undergo significant hypertrophy and accumulation of lipid droplets, particularly in diseased states, that render the cell vulnerable to lysis on cell isolation. Proximity labeling with biotinylating enzymes offers an approach well suited to rapidly and efficiently capture cell‐type specific proteomes in vivo (Branon et al. 2018; Rayaprolu et al. 2022; Hung et al. 2016, 2019; Kim et al. 2014; Kim, Park et al. 2021; Mick et al. 2015; Gingras et al. 2019; Martell et al. 2016; Youn et al. 2018; Price et al. 2021; Kwak et al. 2020; Han et al. 2017; Spence et al. 2019; Cho et al. 2017, 2020; Chan et al. 2019; Li et al. 2020; Pronobis et al. 2021; Lin et al. 2017; Khan et al. 2018; Conlan et al. 2018; Opitz et al. 2017; Wei et al. 2021). In this approach, a promiscuous biotin ligase (e.g., BirA*G3, TurboID) is expressed in a cell‐type dependent manner. On dietary addition of biotin, biotinylating proteins add a biotin moiety at lysine residues within a 10 nm radius of the active enzyme (Branon et al. 2018). Tissue lysis, streptavidin affinity purification and quantitative liquid‐chromatography, tandem mass spectrometry (LC–MS/MS) of peptides derived from biotinylated proteins enable an insight into the proteomic signature of the cell type of interest (Branon et al. 2018). Further, directing the biotinylating enzyme to a specific cellular sub‐compartment can provide insight into compartment specific proteomes. We recently reported on a novel mouse strain allowing conditional activation of a BirA*G3 protein specific to the endoplasmic reticulum (ER) in a cell/tissue type restricted fashion (BirA*G3‐ER) and validated tissue specific labeling of the cell's secretome (Yang et al. 2022). Here, we developed a complementary approach designed to broadly label cytoplasmic proteins and compare cytoplasmic and ER biotinylation profiles of proteins within adipocyte depots in vivo.

## 2 Materials and Methods

*Adapted from Yang et al. (2022).

### 2.1 Animal Studies*

Institutional Animal Care and Use Committees (IACUC) at the University of Southern California reviewed and approved all animal work as performed in this study. All work adhered to institutional guidelines. mESCs (mouse embryo stem cells) (B6(Cg)‐Tyr <c‐2J>/J, Stock no. 000058, The Jackson Laboratory) carrying the loxP‐flanked BirA*G3 were aggregated with 8‐cell C57bl/6J embryos to obtain chimeras. The resulting chimeric mice were bred with R26PhiC31 (B6.129S4‐*Gt*(*ROSA*)*26Sor* ^*tm3* (phiC31^*^)*Sor*^/J) females to remove the attB‐neoR‐attP cassette. The resulting mice that carry the BirA*G3 allele have been deposited at the Jackson laboratories for distribution to the community (JAX stock no. 038060). The *Adipoq‐Cre* mice (B6;FVBTg(Adipoq‐cre)1Evdr/J, strain: 010803, stock no. 028020, The Jackson Laboratory) (Eguchi et al. 2011) were used as previously described. *Adipoq‐Cre* mice crossed to a previously generated BirA*G3‐ER (endoplasmic reticulum localized) mouse strain (Yang et al. 2022) (Gt(ROSA)26Sortm10.1(CAG‐BirA*,‐mKate2)Amc, strain: 037395, JAX stock no. 037395) (Yang et al. 2022) or to the BirA*G3 mice to activate BirA*G3 in mouse adipocytes.

### 2.2 In Vivo Assays*

For all mouse studies, Adipoq‐BirA*G3 and BirA*G3 (control) mice at 8–14 weeks were given biotin via water for 48 h (5 mM, pH 7.4; Sigma B4639‐5G) or chow (2000 ppm; LabDiet, SWLP). For all in vivo assays, tissues were collected as follows. Mice were euthanized at 8–14 weeks; blood was then collected from the inferior vena cava, followed by perfusion with 1X cold DPBS (Dulbecco's Phosphate Buffered Saline). The blood was allowed to clot at room temperature for 30 min and then spun down at 2000 × *g* for 15 min at 4°C. The serum was collected and spun again at 2000 × *g* for 15 min at 4°C, then removed to a fresh tube and flash frozen (in liquid nitrogen) before being stored at −80°C until being used. After perfusion, tissues were collected and rinsed in 1X cold DPBS before being minced with a razor blade and aliquoted into tubes. Tissues were then flash frozen and stored at −80°C until being used.

### 2.3 Frozen Tissue Preparation and Sectioning*

Briefly, tissues were harvested from DPBS‐perfused mice and then fixed in 4% paraformaldehyde overnight at 4°C. Tissues were then washed three times in 1X DPBS with calcium and magnesium before being incubated in 30% sucrose overnight. The next day, tissues were washed in OCT three times to remove excess sucrose and then embedded in OCT (VWR, 25608‐930) and frozen in a dry‐ice ethanol bath before being stored at −80°C. Tissue blocks were thawed to −20°C overnight and then cryosectioned at 10–16 μm at −20°C and placed on glass slides. Slides were then stored at −80°C until processing for immunostaining.

### 2.4 Immunofluorescent Staining and Confocal Microscopy*

Frozen sectioned tissues were thawed at room temperature for 10 min. Slides were then incubated in ice‐cold 50% methanol (VWR BDH1135‐4LG), 50% acetone (VWR AA30698‐K2) to remove lipids. Slides were then rinsed in 1X DPBS with calcium and magnesium for 10 min. Slides were permeabilized in 0.25% Triton‐X100 (Sigma X100‐500ML) for 5 min, then incubated in blocking buffer (2.0% Sea Block (Thermo, 37527) + 0.125% Triton‐X100 in 1X DPBS with calcium and magnesium) for 1 h at room temperature. Slides were then incubated in primary antibody (Table S1) diluted in blocking buffer, overnight at 4°C. The following day, primary antibodies were removed, and slides were washed in blocking buffer four times for 5 min each. Slides were then incubated in secondary antibody diluted in blocking buffer for 1 h at room temperature. Secondary antibody (Table S2) was removed, and slides were washed in blocking buffer four times for 5 min each. Slides were then incubated in 1 mg/mL Hoechst 33342 (Thermo, H3570) in 1X DPBS with calcium and magnesium for 10 min at room temperature. Slides were then washed twice in 1X DPBS with calcium and magnesium for 5 min each. Slides were mounted in Immu‐Mount (Thermo, 9990402) and imaged at 40× or 63× using a Leica SP8 DSLM confocal microscope. Images are average intensity projections from z‐stacks of 0.5–1.0 μm steps. All images presented represent at least three images per tissue and two biological samples per genotype.

### 2.5 Protein Lysate Preparation*

Protein lysates were prepared as described previously (Droujinine et al. 2021; Yang et al. 2022) with the following modifications. Tissues were homogenized in 500 μL RIPA complete lysis buffer (RIPA buffer) (ThermoFisher, 89901) with 1X complete EDTA‐free protease inhibitor cocktail (Sigma, 11873580001), 1 mM benzamidine hydrochloride (VWR, TCB0013‐100G), 4 μM pepstatin A (Sigma, EI10), 100 μM PMSF (Sigma, 11359061001) and bead homogenized using stainless steel beads (NextAdvance, SSB14B‐RNA) for 5 min at setting 10 in a Bullet Blender Storm (NextAdvance, BT24M) model. Samples were then centrifuged at 14,000 × *g* for 15 min at 4°C. Supernatants were transferred to protein loBind (Eppendorf) tubes. Protein lysate concentrations were determined using Pierce BCA (Thermo, 23227) microplate assay per manufacturer's instructions. Lysates were then stored at −80°C.

### 2.6 Streptavidin Beads Pulldowns*

Streptavidin pulldowns were performed as described previously (Droujinine et al. 2021; Yang et al. 2022) with modifications. Streptavidin magnetic beads (Thermo, 88817) or high‐capacity streptavidin agarose (Thermo 20359) beads were resuspended in lysis buffer (above) by beads packed by magnetic separation (BioRad 1614916) or by centrifugation. We tested a series of volumes of beads and washing conditions and found that 20 μL beads per 100 μg protein for adipose tissue and 60 μL of high‐capacity streptavidin agarose beads (Thermo 20359) with 4 mg protein together with the following washing conditions are optimal. Pulldown reactions were set up in 450 μL lysis buffer with bead:protein ratio determined above. Affinity purifications were then incubated overnight at 4°C in a wheel rotator. The following day, affinity purification reactions were washed twice in lysis buffer, then once in 8 M Urea (Sigma U5378‐500G) in 100 mM Tris (pH 8.5), and finally twice in lysis buffer. After the final wash, lysis buffer was removed and beads were either boiled in 12 μL 1X loading buffer (Li‐Cor 928‐40004) with 1.43 M β‐mercaptoethanol (1:10) or resuspended in 100 μL lysis buffer and flash frozen for western blotting and mass spectrometry, respectively.

### 2.7 Fluorescent Western Blot Analysis*

Western blots were performed with standard protocols and the following modifications. Equal amounts of total protein lysate were loaded per sample per reaction with 1X Li‐Cor loading buffer (Li‐Cor, 928‐40004) with 1.43 M β‐mercaptoethanol. For streptavidin pulldowns, beads were resuspended in 12 μL 1X Li‐Cor loading buffer (Li‐Cor, 928‐40004) with 1.43 M β‐mercaptoethanol. All samples were then boiled at 95°C for 5 min to elute, then briefly spun down and kept on ice prior to loading. Total protein samples and pulldown elutes were loaded on 10% SDS tris‐glycine acrylamide gels and ran in standard 1X SDS‐Tris‐glycine Running buffer with Li‐Cor 5 μL one‐color molecular marker (Li‐Cor, 928‐40000) at 60 V for 30 min, followed by 120 V for 50 min. Samples on the gel were transferred to methanol activated PVDF 0.45 μm membranes using BioRad's wet tank mini‐protean system for 1–3 h at 250–300 constant mA in a sample dependent context or by semi‐dry transfer using a BioRad Trans‐Blot system (BioRad, 174150) transfer conditions ranges from 12 to 30 min depending upon protein (available upon request). After transfer, membranes were dried at 37°C for 5 min and then re‐activated with methanol. Blots were stained with Li‐Cor's Revert‐700 Total Protein Stain (Li‐Cor, 926‐11010) for normalization and imaged using a Li‐Cor Odyssey Clx. Blots were then de‐stained per kit instructions and put in block (Li‐Cor Intercept block, 927‐60001) for 1 h, room temperature, shaking. Blots were then transferred to primary antibody (Table S1) (block with 0.2% Tween20) overnight at 4°C, shaking. The following day, blots were washed four times in TBS‐T for 5 min each at room temperature, shaking, and then incubated in secondary antibody (Table S2) in block with 0.2% Tween20 and 0.1% SDS, and/or streptavidin conjugate (1:5000; 680 or 800, Li‐Cor, 926‐68079, 926‐32230) if visualizing biotinylated proteins, for 1 h at room temperature, shaking. Blots were then washed twice with TBS‐T for 5 min each, room temperature, shaking, followed by two 5‐min TBS washes at room temperature, shaking. Blots were imaged on a Li‐Cor Odyssey Clx using Li‐Cor's ImageStudio (Version 5.2.5). After imaging blots were dried at 37°C for 5 min, then stored. All western blot images were exported from Li‐Cor, pseudo‐colored and converted to RGB tiffs in ImageJ (v1.51S) for figures. For specific proteins, bands were selected based on molecular weight from antibody manufacturer information and literature.

### 2.8 Sample Digestion

Streptavidin‐bound proteins were reduced and alkylated on bead via sequential 20‐min incubations with 5 mM TCEP and 10 mM iodoacetamide at room temperature in the dark while being mixed at 1200 rpm in an Eppendorf thermomixer. Proteins were then digested by the addition of 0.1 μg Lys‐C (NEB, P8109S) and 0.8 μg Trypsin (Thermo Scientific, 90057) while shaking at 37°C overnight.

### 2.9 TMT Labeling and CIF Fractionation

The supernatant was transferred to new tubes, and 12 μL of carboxylate‐modified magnetic beads (CMMB, and also widely known as SP3 (Hughes et al. 2014)) was added to each sample. A 100% acetonitrile was added to each sample to increase the final acetonitrile concentration to > 95% and induce peptide binding to CMMB. CMMB was then washed three times with 100% acetonitrile and then resuspended with TMT labeling buffer. 50ug of each sample was labeled using TMT10plex (Thermo Fisher Scientific, 90113) and the labeled samples were pooled. The pooled sample was fractionated by the CMMB‐based Isopropanol Gradient Peptide Fractionation (CIF) method (Deng et al. 2021) into three fractions before MS analysis.

### 2.10 LC–MS Acquisition and Analysis

Fractionated samples were separated on a 75 μm ID × 25 cm C18 column packed with 1.9 μm C18 particles (Dr. Maisch GmbH) using a 140‐min gradient of increasing acetonitrile and eluted directly into a Thermo Orbitrap Fusion Lumos mass spectrometer where MS spectra were acquired using SPS‐MS3.

Protein identification was performed using MaxQuant (Cox and Mann 2008) v 1.6.17.0. The complete Uniprot mouse proteome reference database (UP000000589) was searched for matching MS/MS spectra. Searches were performed using a 20 ppm precursor ion tolerance. TMT10plex was set as a static modification on lysine and peptide N terminal. Carbamidomethylation of cysteine was set as a static modification, while oxidation of methionine residues and N‐terminal protein acetylation were set as variable modifications. LysC and Trypsin were selected as enzyme specificity with a maximum of two missed cleavages allowed. A 1% false discovery rate was used as a filter at both protein and PSM levels.

Statistical analysis was conducted with the MSstatsTMT Bioconductor package (Huang et al. 2020). The abundance of proteins missing from one condition but found in more than two biological replicates of the other condition for any given comparison was estimated by imputing intensity values from the lowest observed reporter ion intensity across samples, and *p*‐values were randomly assigned to those between 0.05 and 0 for illustration purposes.

### 2.11 Data Analysis and Statistics

Data was analyzed using Microsoft Excel, R (version 4.1.3 (2022‐03‐10)), and RStudio (version 2022.02.1 + 461 “Prairie Trillium” Release (8aaa5d470dd82d615130dbf663ace5c7992d48e3, 2022‐03‐17) for macOS Mozilla/5.0 (Macintosh; Intel Mac OS X 12_2_1) AppleWebKit/537.36 (KHTML, like Gecko) QtWebEngine/5.12.10 Chrome/69.0.3497.128 Safari/537.36). For secretion annotations, proteins were annotated based on the subcellular localization data from UniProt and the cellular component data from the National Center for Biotechnology Information (NCBI) (https://www.ncbi.nlm.nih.gov). Proteins in fasta formats were uploaded to SignalP 6.0 (https://services.healthtech.dtu.dk/service.php?SignalP‐6.0) and TMHMM (v 2.0) (https://services.healthtech.dtu.dk/service.php?TMHMM‐2.0) for the prediction of SignalP and transmembrane helix, separately. Gene ontology function annotation was performed using EnrichGO in clusterProfiler (3.16.1). The top GO terms were visualized by bar plots in ggplot2. PCA was used to study the similarities between samples. The analysis was conducted without filtering any proteins.

## 3 Results

### 3.1 Generation of Proximity Labeling Mice

**FIGURE 1:**
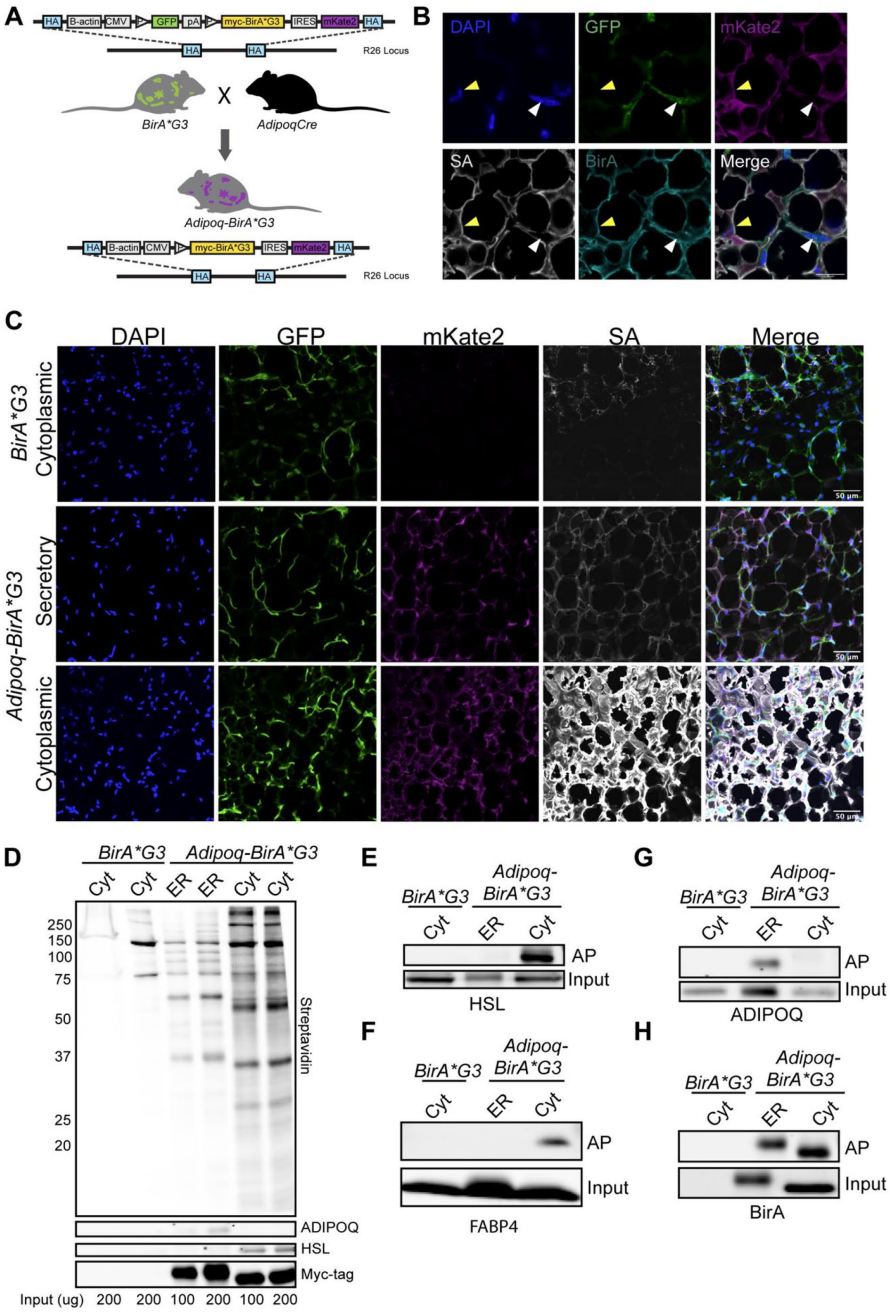
Adipocyte‐specific in vivo proximity labeling. (A) Schematic of adipocyte‐specific proximity labeling. Cytoplasmic proximity labeling mice were crossed to adiponectin‐Cre mice to generate adipocyte proximity labeling mice (Adipoq‐BirA*G3). (B) Immunofluorescent staining shows expression of native GFP, native mKate2, BirA*G3, and biotinylated proteins (SA: streptavidin) in posterior adipose tissue sections of Adipoq‐BirA*G3 mice. Yellow arrows: GFP‐/mKate2+ nuclei, White arrows: GFP+/mKate2‐ nuclei; Scale Bar: 10 μm. (C) Immunofluorescent staining shows expression of native GFP, native mKate2, BirA*G3, and biotinylated proteins (streptavidin: SA) in posterior adipose tissue sections of Adipoq‐BirA*G3‐ER, Adipoq‐BirA*G3, and BirA*G3 (control). Scale Bar: 50 μm. (D) Western blot of streptavidin affinity purified proteins from posterior adipose tissue in Adipoq‐BirA*G3 mice compared to Adipoq‐BirA*G3‐ER mice and BirA*G3 (control) mice. Each sample is from an individual mouse (*n* = 1/genotype) with *n* = 2 technical replicates per sample. HSL, BirA (myc‐tag); Lower, Adiponectin (ADIPOQ); Upper, streptavidin. Input: 100 or 200 μg. (E–H) Western blot of streptavidin affinity purified (AP) proteins and input (total) protein lysate from posterior adipose tissue in Adipoq‐BirA*G3 mice compared to Adipoq‐BirA*G3‐ER mice and BirA*G3 (control) mice. Each lane is from an individual mouse (*n* = 1/genotype). AP Input: 200 μg. Input bands: 20 μg.

We recently generated a mouse model (BirA*G3‐ER) to study cell‐type specific secretion through BirA*G3‐ER‐mediated biotinylation of proximal proteins (Yang et al. 2022). In this model, cell type specific Cre recombinase activity enabled production of a secreted myc‐tagged BirA*G3 that was retained within the endoplasmic reticulum (ER) through a C‐terminal ER retention sequence (KDEL) (Yang et al. 2022). To complement this approach, we used an identical recombination approach into the ROSA 26 safe harbor locus to create a Cre recombination dependent BirA*G3 mouse strain (lacking a signal peptide) to allow for broad protein biotinylation within the cell's cytoplasm (Figure 1A). Prior to Cre‐mediated recombination, a *CAGGS* regulatory sequence drives constitutive expression of a GFP reporter that blocks downstream transcription of BirA*G3 and a transcriptionally linked mKate2 reporter (Droujinine et al. 2021; Yang et al. 2022). Cre‐mediated recombination excises the GFP cassette, resulting in expression of myc‐tagged BirA*G3 and mKate2 (Figure 1A).

To study adipocyte‐specific proteomes, Adipoq‐BirA*G3 and BirA*G3‐ER mice were generated to compare cytoplasmic and secreted adipocyte profiles. Initially, we verified expected outcomes with the newly generated BirA*G3 allele. As expected, Cre‐mediated recombination within adipocytes led to the production of BirA*G3 and mKate2, while non‐adipocytes were GFP^+^, mKate2^−^, and BirA*G3^−^ (Figure 1B), as observed for Adipoq‐BirA*G3‐ER mice (Figure 1C). BirA*G3 and mKate2 co‐localized with adipocyte markers (HSL) supporting adipocyte‐specific allele recombination (Figure S1). The lymph node (LN) within subcutaneous adipose tissue showed a clear border with BirA+/mKate2+ adipocytes and was GFP+, mKate2‐, and BirA‐, further supporting adipocyte‐specific recombination and BirA activity (Figure S1). Biotinylation of adipocytes in Adipoq‐BirA*G3 mice was greatly increased compared to the Adipoq‐BirA*G3‐ER samples, though adipocytes showed similar levels of BirA*G3 and BirA*G3‐ER protein consistent with broader cellular activity and a greater spectrum of target proteins for the non‐secreted enzyme (Figure 1B–D). Streptavidin affinity purification of biotinylated proteins from subcutaneous adipose tissue (SAT) confirmed the enhanced biotinylation of target proteins in Adipoq‐BirA*G3 to Adipoq‐BirA*G3‐ER, both of which showed markedly elevated biotinylation relative to unrecombined (control) alleles (Figure 1C,D). Comparison of streptavidin affinity purified biotinylated proteins from the SAT of Adipoq‐BirA*G3 and Adipoq‐BirA*G3‐ER mice detected adiponectin (ADIPOQ), a secreted adipokine, in the Adipoq‐BirA*G3‐ER mice, and HSL and FABP4, two predominantly cytoplasmic proteins in the Adipoq‐BirA*G3 mice, supporting expected cellular compartment restricted activity for BirA*G3‐ER and BirA*G3 in adipocytes (Figure 1D–H).

### 3.2 Sex and Depot Specific Differences in the Adipocyte Proteome

**FIGURE 2:**
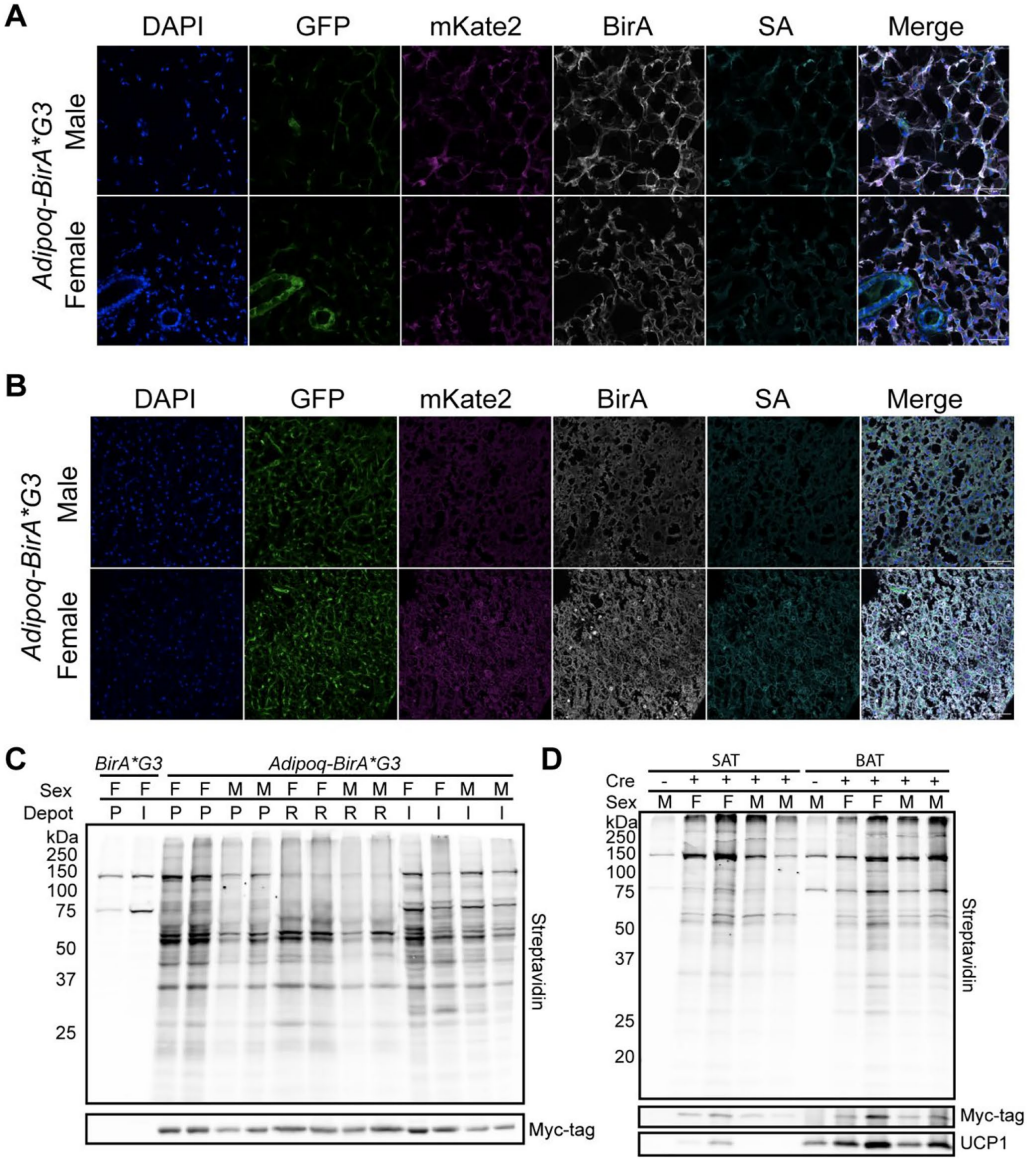
Expression and activity of BirA*G3 across depots and sex in cytoplasmic proximity labeling mice. (A) Immunofluorescence staining shows expression of native GFP, native mKate2, BirA*G3, and biotinylated proteins (SA: streptavidin) in posterior subcutaneous adipose tissue sections from male and female Adipoq‐BirA*G3 mice. Scale Bar: 50 μm. (B) Immunofluorescence staining shows expression of native GFP, native mKate2, BirA*G3, and biotinylated proteins (SA: streptavidin) in interscapular brown adipose tissue sections from male and female Adipoq‐BirA*G3 mice. Scale Bar: 50 μm. (C) Western blot of total protein lysate from posterior (P, white, subcutaneous), retroperitoneal (R, white, visceral), and interscapular (I, brown) adipose tissue in male (M) and female (F) Adipoq‐BirA*G3 mice and female BirA*G3 (control) mice. Each lane is from an individual mouse (*n* = 4/depot, *n* = 2/sex in Adipoq‐BirA*G3 (Cre+) condition). Input: 25 μg. Lower, BirA (Myc‐tag, ~35 kDa); Upper, streptavidin. (D) Western blot of streptavidin affinity purified proteins from posterior (P, white, subcutaneous) and interscapular (I, brown) adipose tissue in male and female Adipoq‐BirA*G3 mice. Each lane is from an individual mouse (*n* = 4/depot, *n* = 2/sex in Adipoq‐BirA*G3 (Cre+) condition). Input: 100 μg. Lower: BirA (Myc‐tag, ~35 kDa); UCP1 (~33 kDa); Upper: streptavidin.

We utilized the Adipoq‐BirA*G3 mouse line to compare SAT and BAT depots between the sexes. Immunofluorescence of posterior SAT and interscapular BAT from males and females showed a variance in adipocyte size marked by BirA*G3 and SA (Figure 2A,B). Western blot analysis of posterior SAT, retroperitoneal visceral (VAT), and interscapular BAT depots showed differences in protein biotinylation intensity between male and female mice (Figure 2C,D). Interscapular BAT, composed of noticeably smaller, multilocular lipid droplets, was distinguished by the brown adipocyte marker, uncoupling protein 1 (UCP1; Figure 2D, Figure S2A). As expected, UCP1 was detected in total protein lysate and streptavidin affinity purification western blot analysis in the interscapular BAT depot (Figure 2D). Interestingly, UCP1 was also detected in the total protein lysate and after streptavidin affinity purification in the SAT depot of female Adipoq‐BirA*G3 mice (Figure 2D), suggesting an enhanced representation of beige adipocytes in posterior adipose tissue of the female (Elmasri et al. 2009).

### 3.3 MS Profiling and Quantitation of Adipocyte Depot‐Specific Proteomes

**FIGURE 3:**
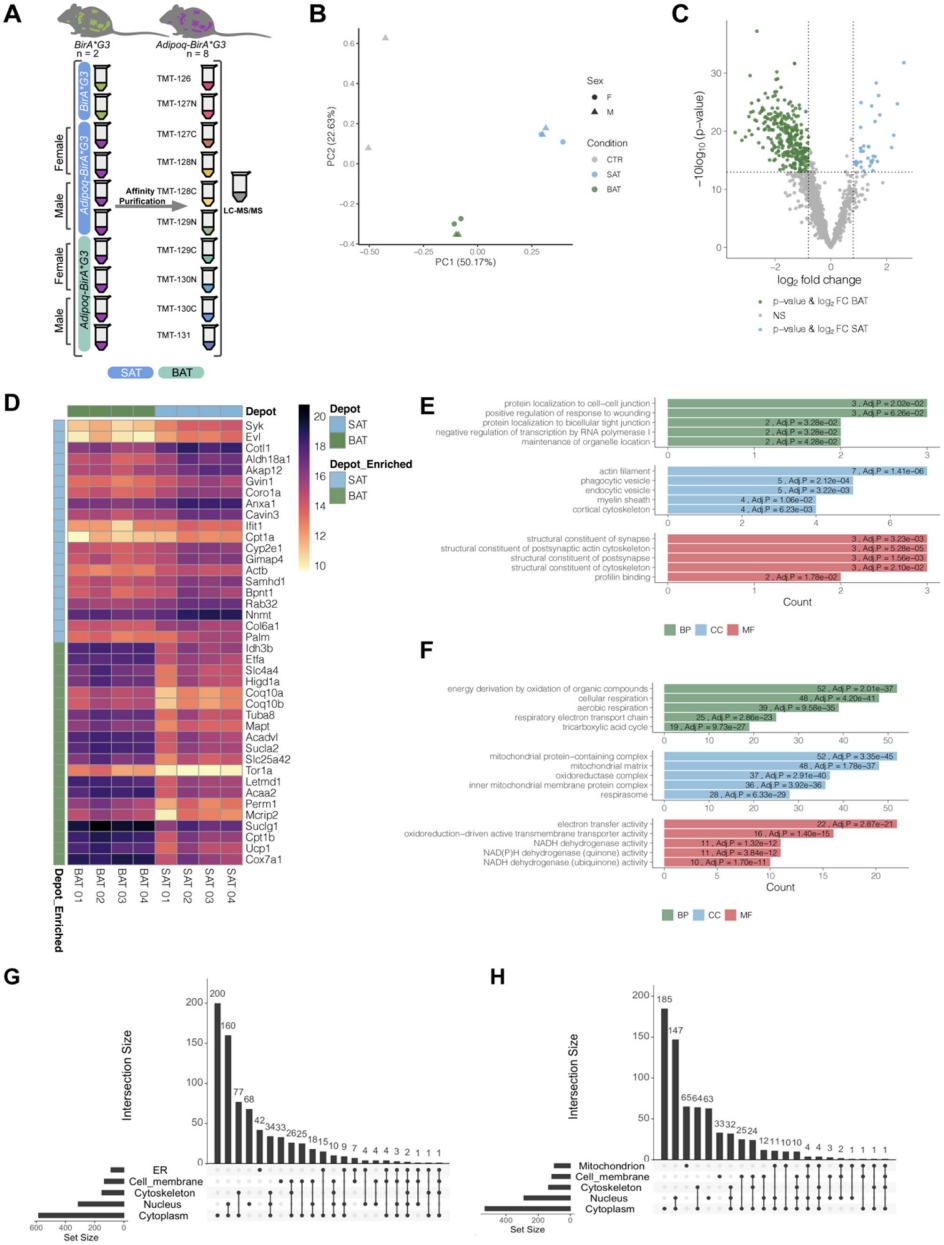
Detection and proximity labeling based proteome profiling of brown and white adipocytes. (A) Tandem Mass Tag (TMT)‐based 10plex LC–MS/MS workflow to identify adipocyte proteome differences in males and females between posterior subcutaneous white (SAT) and interscapular brown (BAT) adipose tissue of Adipoq‐BirA*G3 (*n* = 8) and BirA*G3 (*n* = 2) mice. (B) Principal component analysis of streptavidin affinity purified proteins in SAT and BAT. Each point represents one biological sample (SAT Adipoq‐BirA*G3 in blue, BAT Adipoq‐BirA*G3 in green, and BirA*G3 controls in gray; circle: F, female, triangle: M, male). (C) Volcano plot of proteins detected in Adipoq‐BirA*G3 mice from interscapular BAT and posterior SAT depots. Log_2_FC plotted on the *x*‐axis, −10log_10_(*p*‐value) plotted on the *y*‐axis. Significantly enriched proteins (*p*‐value < 0.05, log_2_FC > 1.0) shown in blue (SAT) and green (BAT). (D) Heatmap of top 20 depot‐specific significantly enriched proteins by log_2_FC from panel C. (E) Gene ontology (GO) term analysis for enriched proteins related to biological process (BP; green), cellular compartment (CC; blue), and molecular function (MF; red) for significantly enriched posterior SAT adipocyte proteins in Adipoq‐BirA*G3 mice. (F) Gene ontology (GO) term analysis for enriched proteins related to biological process (BP; green), cellular compartment (CC; blue), and molecular function (MF; red) for significantly enriched interscapular BAT adipocyte proteins in Adipoq‐BirA*G3 mice. (G) Upset plot subcellular localization by Uniprot annotations of enriched proteins (*p*‐value < 0.05, log_2_FC > 1.0) from Adipoq‐BirA*G3 subcutaneous adipose tissue over BirA*G3 (control) subcutaneous adipose tissue. Upset plot shows protein distribution among annotations to single compartments or multiple compartments. (H) Upset plot subcellular localization by Uniprot annotations of enriched proteins (*p*‐value < 0.05, log_2_FC> 1.0) from Adipoq‐BirA*G3 brown adipose tissue over BirA*G3 (control) brown adipose tissue. Upset plot shows protein distribution among annotations to single compartment or multiple compartments.

To profile BAT and SAT proteomes from male and female mice, affinity‐purified biotinylated proteins were analyzed by quantitative TMT LC–MS/MS (Figure 3A). Principal component analysis (PCA) showed discrete clustering of samples by depot and genotype (Figure 3B). The majority of proteins were enriched in the Adipoq‐BirA*G3 BAT and SAT samples compared to the BirA*G3 (CTR; control) samples: 1323 BAT/CTR and 1226 SAT/CTR enriched proteins (log_2_ fold change (FC) > 1.0 and *p*‐value < 0.05; Figures S3A,B, S4C, S5C). Depot‐specific enrichment analysis identified 299 BAT and 35 SAT enriched proteins (log_2_ FC > 1.0 (BAT) or log_2_ FC < −1.0 (SAT) and *p*‐value < 0.05; Figure 3C). Comparing samples within a genetic sex grouping identified 20 BAT and 23 SAT enriched proteins in males and 103 BAT and 4 SAT enriched proteins in females (Figures S4C and S5C). Volcano plot analysis highlighted enrichment of posterior SAT and interscapular BAT proteins in males and females (Figure 3C, Figures S4B and S5B). Examining the levels of depot‐specific proteins between biological replicates showed consistency among replicates in a heatmap analysis (Figure 3D, Figures S4D, S5D). As expected, UCP1, a known BAT marker (Lee and Fried 2017), was significantly enriched in BAT Adipoq‐BirA*G3 samples compared to SAT Adipoq‐BirA*G3 samples (log_2_FC 3.18 BAT/SAT) and was the second most BAT enriched protein (Figure 3D). Western blot analysis further confirmed UCP1 BAT enrichment after streptavidin affinity purification and showed even BirA (log_2_FC 0.14 BAT/SAT) levels between SAT and BAT samples (Figure S3E–G). Further, gene ontology (GO) analysis highlighted terms related to mitochondrial function and respiration as over‐represented in BAT versus SAT comparison (Figure 3E,F, Figures S4E, S5E). Protein compartment annotation of identified proteins showed the majority of Adipoq‐BirA*G3 labeled proteins were annotated to the cytoplasm, though multiple cellular compartments were predicted (Figure 3G,H, Figure S3C,D).

### 3.4 MS Identification Sex‐Specific Adipocyte Proteins

**FIGURE 4:**
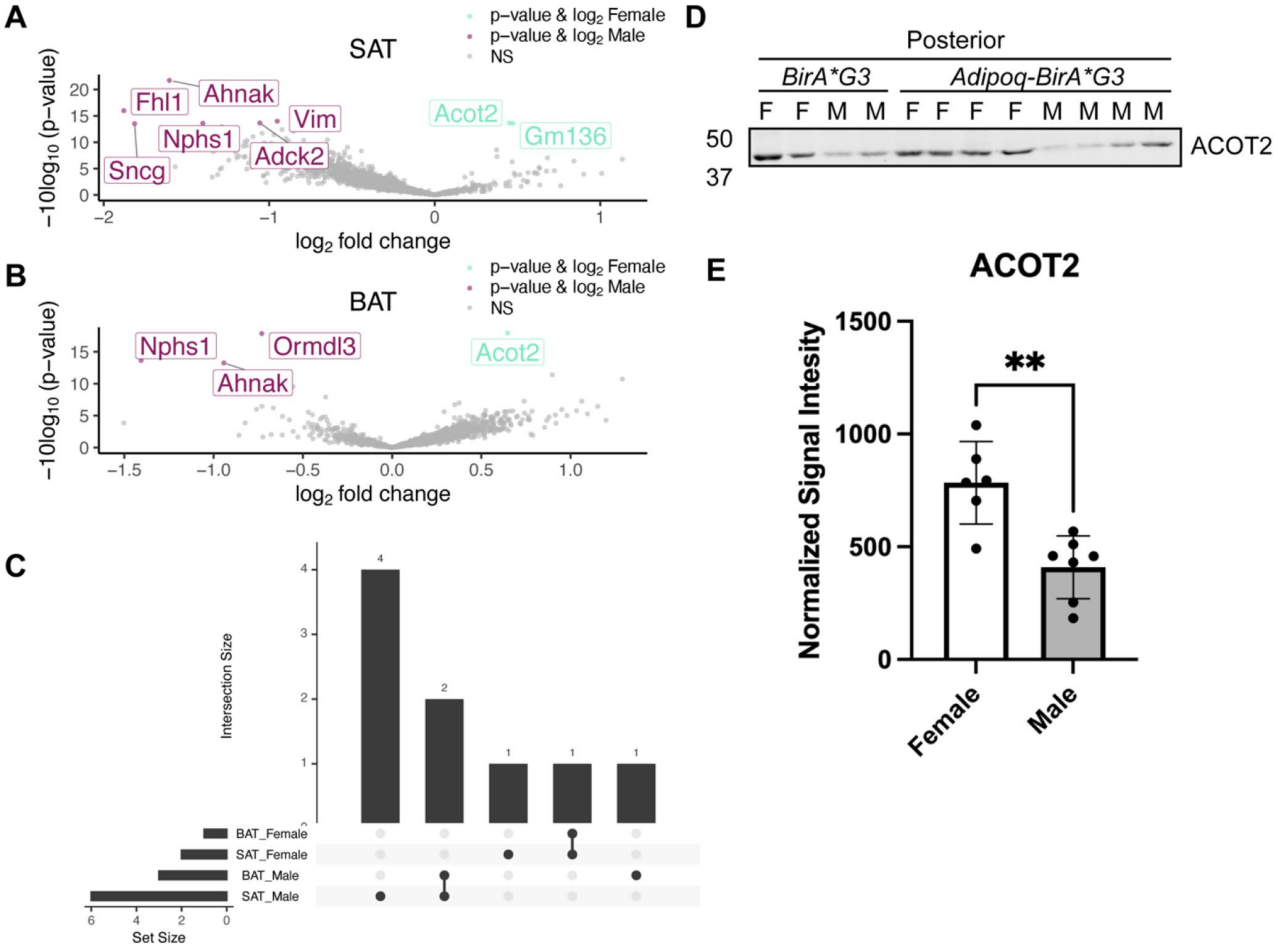
Detection and proteome profiling of sex‐specific adipocyte proteins. (A) Volcano plot of proteins detected in Adipoq‐BirA*G3 mice from male and female mice in SAT depots. Log_2_FC is plotted on the *x*‐axis, −10log_10_(*p*‐value) plotted on the *y*‐axis. Significantly enriched proteins (*p*‐value < 0.05, log_2_FC> 0) shown in teal (female) and pink (male). (B) Volcano plot of proteins detected in Adipoq‐BirA*G3 mice from male and female mice in BAT depots. Log_2_FC plotted on the *x*‐axis, −10log_10_(*p*‐value) plotted on the *y*‐axis. Significantly enriched proteins (*p*‐value < 0.05, log_2_FC> 0) shown in teal (female) and pink (male). (C) Upset plot analysis indicates 4 enriched proteins in male SAT, 1 enriched protein in male BAT, 2 enriched proteins in both male SAT and BAT, 1 enriched protein in female SAT, and 1 enriched protein in both female SAT and BAT. (D) Western blot of total protein lysate (T) from subcutaneous posterior adipose tissue in male (M, *n* = 6) and female (F, *n* = 6) Adipoq‐BirA*G3 mice and BirA*G3 (control) mice. (*n* = 4/genotype in Cre + condition) Input: 25 μg. ACOT2. Total protein stain used for normalized loading in Figure S6A. (E) Normalized signal intensity (Figure S6B) of ACOT2 in Figure 4D between males (*n* = 6) and females (*n* = 6) in subcutaneous posterior adipose. **, significant; *p*‐value, 0.0013 (Welch's one‐tailed *t*‐test).

To attempt to identify differences in the BAT and SAT proteomes between male and female mice, affinity purified biotinylated proteins were analyzed by quantitative TMT mass spectrometry. Only a small number of significant differences were observed between the sexes. Three male BAT (NPHS1, AHNAK, ORMDL3) and six male SAT (FHL1, SNCG, AHNAK, NPHS1, VIM, ADCK22) enriched proteins were identified and one female BAT (ACOT2) and two female SAT (ACOT2, *Gm136*) enriched proteins (*p*‐value < 0.05;Figure 4A–C). Of these, ACOT2 was enriched in females in both BAT and SAT, while NPHS1 and AHNAK were enriched in males in both BAT and SAT (Figure 4C). We validated ACOT2 by western blot analysis, which further confirmed significant enrichment of ACOT2 in female BAT and SAT extracts (Figure 4D,E).

## 4 Discussion

Here, we report on a mouse strain for CRE‐dependent, cell type‐specific in vivo biotinylation of proteins, illustrating the utility of the strain by investigating the adipocyte proteome. Adipocyte‐specific proximity labeling and affinity purification successfully captured and identified adipocyte proteins.

Specifically, BirA*G3 intracellular proximity labeling successfully biotinylated numerous adipocyte proteins, including those known to be predominantly localized to the cytoplasm (HSL, FABP4) (Elmasri et al. 2009; Althaher 2022). Depot‐specific profiling captured 299 BAT and 35 SAT enriched proteins, which showed depot‐specific signatures such as mitochondrial and respiration signatures in BAT. Of the 299 proteins identified in BAT, there was a strong enrichment for mitochondrial proteins, and gene ontology analysis showed further enrichment of mitochondrial function. Interestingly, our study identified significantly more proteins enriched in BAT compared to SAT. Another study investigating proteomic signatures of BAT and SAT also identified numerous BAT enriched proteins (318) while only identifying single proteins for SAT (Müller et al. 2016). Comparison of enriched BAT proteins between these studies showed overlap of known BAT proteins, including UCP1 and VDAC2 (Müller et al. 2016). Of the 35 SAT proteins identified here, there was not a strong signature of white adipose tissue, and gene ontology analysis showed enrichment for terms related to the cytoskeleton, protein localization to cell–cell junctions, and endocytic vesicles. However, when compared to control samples, we did see enrichment of known white adipocyte proteins such as FABP4, LPL, FASN, ISR‐1, PLIN1, and HSL, suggesting that although we are enriching white adipose tissue proteins, we do not see strong enrichment when compared to brown adipose tissue. Overall, we identified enriched proteins primarily in BAT, and these significantly enriched adipocyte proteins identified through proximity labeling approaches further contribute to studies in adipocyte depot differences.

Comparison of male and female adipose tissue proteomes resulted in few, though potentially relevant, sex‐specific differences for male and female enriched proteins. ACOT2 (acyl‐coenzyme A thioesterase 2, a mitochondrial localized protein) was enriched in both female adipose tissue depots compared to males. ACOT2 plays a central role in fatty acid metabolism in the mitochondria by hydrolyzing esters in coenzyme A (CoA) to generate free fatty acid and CoA (Bekeova et al. 2019; Moffat et al. 2014). ACOT2 is reported to be up‐regulated in thermogenic adipocytes (brown/beige) (Vijay et al. 2015; Cruciani et al. 2022) and increased ACOT2 gene expression has been observed in female rat hearts (Vijay et al. 2015), suggesting ACOT2 could be sexually dimorphic in another tissue. Upregulation of ACOT2 in females, in conjunction with greater expression of UCP1, suggests that increased thermogenesis is acting as a protection against metabolic disease (Vijay et al. 2015; Cruciani et al. 2022). Future studies investigating the role of ACOT2 in brown adipocytes could provide insights on how to modulate its gene expression to increase BAT in males. In male mice, AHNAK was enriched in both BAT and SAT. AHNAK plays a role in calcium channel modulation and homeostasis (Lee et al. 2014; Sundararaj et al. 2021). AHNAK nucleoprotein, desmoyokin, knockout mice have strong resistance to high fat diet induced obesity and a deficiency of AHNAK promotes browning and thermogenetic gene expression in white adipose tissue (Kim, Shin et al. 2021; Shin et al. 2016). As males are more susceptible to metabolic diseases, AHNAK could be a potential sexually dimorphic protein in the male risk for obesity if the mouse findings here are corroborated in human tissue. Future studies focused on how AHNAK levels and its modulation may relate to diet induced obesity to help determine if AHNAK could be a therapeutic target for metabolic syndrome. Additional studies are required to secondarily validate findings here and provide functional insight into the potential sex‐biased activities of these proteins in mammalian adipocytes.

Several mouse models have been reported employing proximity labeling strategies (Rayaprolu et al. 2022; Droujinine et al. 2021; Yang et al. 2022; Pronobis et al. 2021). The first of these, the BioID2 mouse, labels intracellular components similar to the strain reported here, but with lower labeling efficiency reflecting reduced activity of BioID2 compared to BirA*G3 (Liu et al. 2021; Feng et al. 2020). Another group reported a strain with similar cellular localization generated with the TurboID variant of BirA (Rayaprolu et al. 2022). While TurboID has higher activity than BirA*G3 in biotin‐supplemented conditions, BirA*G3 has a higher affinity for biotin and greater activity in the presence of endogenous biotin (Branon et al. 2018). The TurboID system lacks the dual reporter in the BirA*G3 mouse reported here that allows for rapid and efficient characterization of allele recombination and activity. The same reporter system is employed in our previous BirA*G3‐ER secretome mouse that specifically biotinylates proteins within the secretory pathway (Yang et al. 2022).

Although proximity labeling offers a new approach to study cell‐type specific proteomes, there are limitations. Methods are still being established to best identify protein candidates from the large swath of proteins identified. Additionally, mass spectrometry has its drawbacks, two key ones for proximity labeling being false positives in peptide to protein mapping and the relatively sparse profiling of the sample with related issues of sensitivity, coverage, and quantification. These concerns highlight the importance of secondary approaches to validate key findings and recognition of a likely substantial set of false negative data comparing proteins across studies.

In conclusion, this study validates key expectations of the genetic model through a focus on adipocytes as a target of interest, identifying sex and depot differences in adipocyte proteomes. The CAGGS‐BirA*G3 and CAGGS‐BirA*G3‐ER strains provide powerful complementary community resources for cell type and cell compartment specific proximity labeling in the mouse. Both strains are available through the Jackson Laboratories (see Section 2).

## Author Contributions

T.L.S., A.S.M., and A.P.M. designed experiments. T.L.S., A.S.M., J.G., J.S., J.A.W., I.A.D., and N.P. performed experiments, data collection, and/or data analysis. T.L.S., A.S.M., and A.P.M. wrote the manuscript.

## Disclosure

A.P.M. receives consulting fees or stock options for his scientific advisory role for eGENESIS, TRESTLE BioTherapeutics, and IVIVA Medical.

## Conflicts of Interest

The authors declare no conflicts of interest.

## Supporting information


**Data S1.** Supplementary Figures and Tables.

## Data Availability

The original mass spectra and the protein sequence databases used for searches have been deposited in the public proteomics repository MassIVE (https://massive.ucsd.edu/ProteoSAFe/static/massive.jsp) MS data and analysis scripts are available on Github (MS data and analysis scripts are available on Github (https://github.com/asmeyer/Sex‐and‐Depot‐Specific‐Adipocyte‐Proteome‐Profiling‐in‐vivo‐via‐Intracellular‐Proximity‐Labeling.git)). The following public databases were used: Uniprot (https://www.uniprot.org), mouse (https://www.uniprot.org/proteomes/UP000000589), SignalP 6.0 (https://services.healthtech.dtu.dk/service.php?SignalP‐6.0), TMHMM 2.0 (https://services.healthtech.dtu.dk/service.php?TMHMM‐2.0). Corresponding authors will provide original data upon request. Source data are provided with this paper.
