# Supplementary material for: Sex and Depot Specific Adipocyte Proteome Profiling In Vivo via Intracellular Proximity Labeling
Source: Compr Physiol. 2025 Apr 3;15(2):e70007. doi: 10.1002/cph4.70007 (PMC11969033; doi:10.1002/cph4.70007)
Supplement: Supplementary file 1 — Data S1. Supplementary Figures and Tables. [file CPH4-15-e70007-s001.docx]

# SUPPLEMENTAL MATERIAL


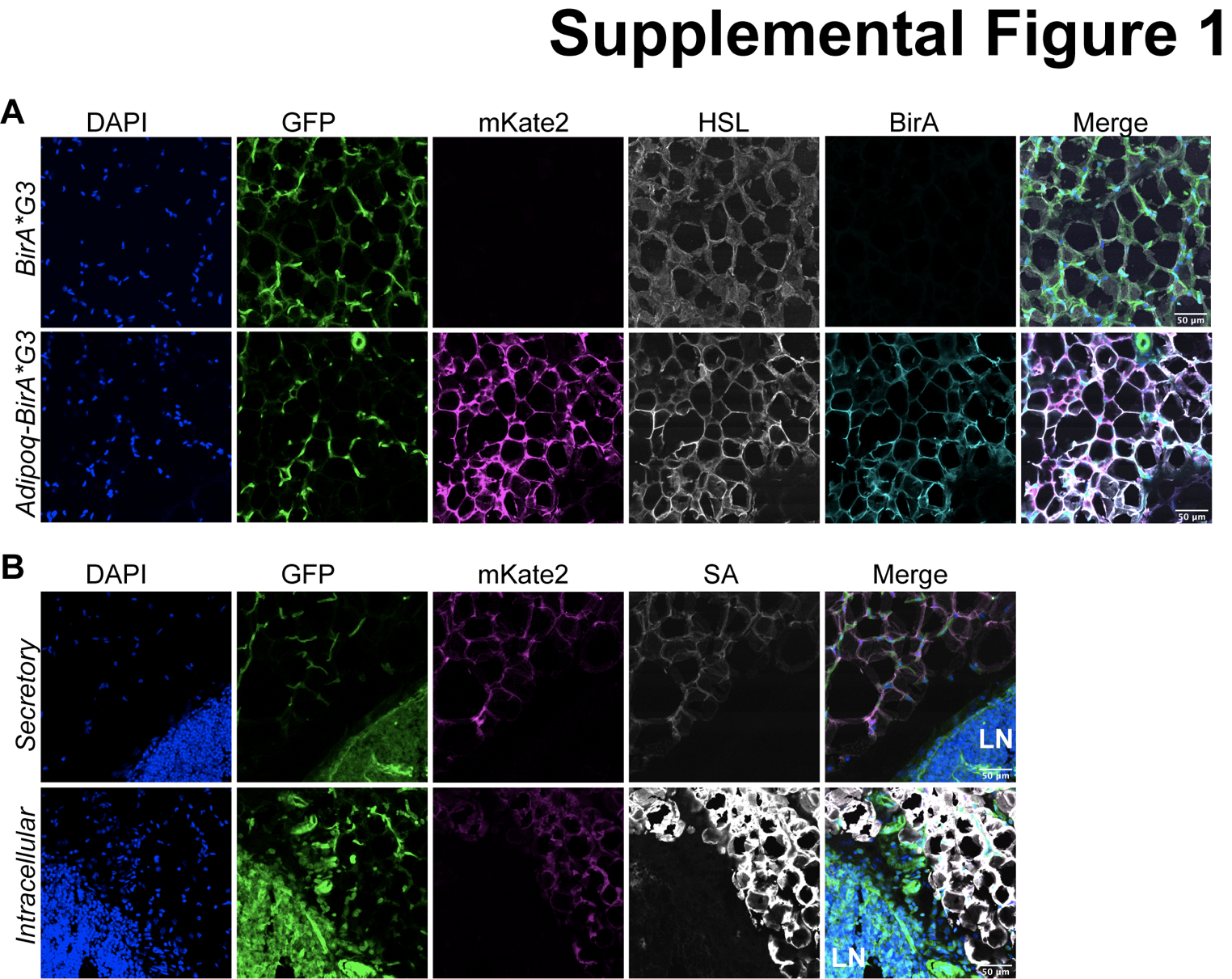


***Supplemental Figure 1. BirA allele recombination is restricted to adipocytes.***

*(A) Immunofluorescence staining shows expression of native GFP, native mKate2, BirA, and HSL (adipocyte marker protein) in posterior subcutaneous adipose tissue sections from Adipoq-BirA*G3 and BirA*G3 mice.*

*(B) Immunofluorescence staining shows expression of native GFP, native mKate2, BirA, and biotinylated proteins (SA: streptavidin) in the posterior subcutaneous adipose tissue sections from Adipoq-BirA*G3 and Adipoq-BirA*G3-ER mice. LN: lymph node*

**
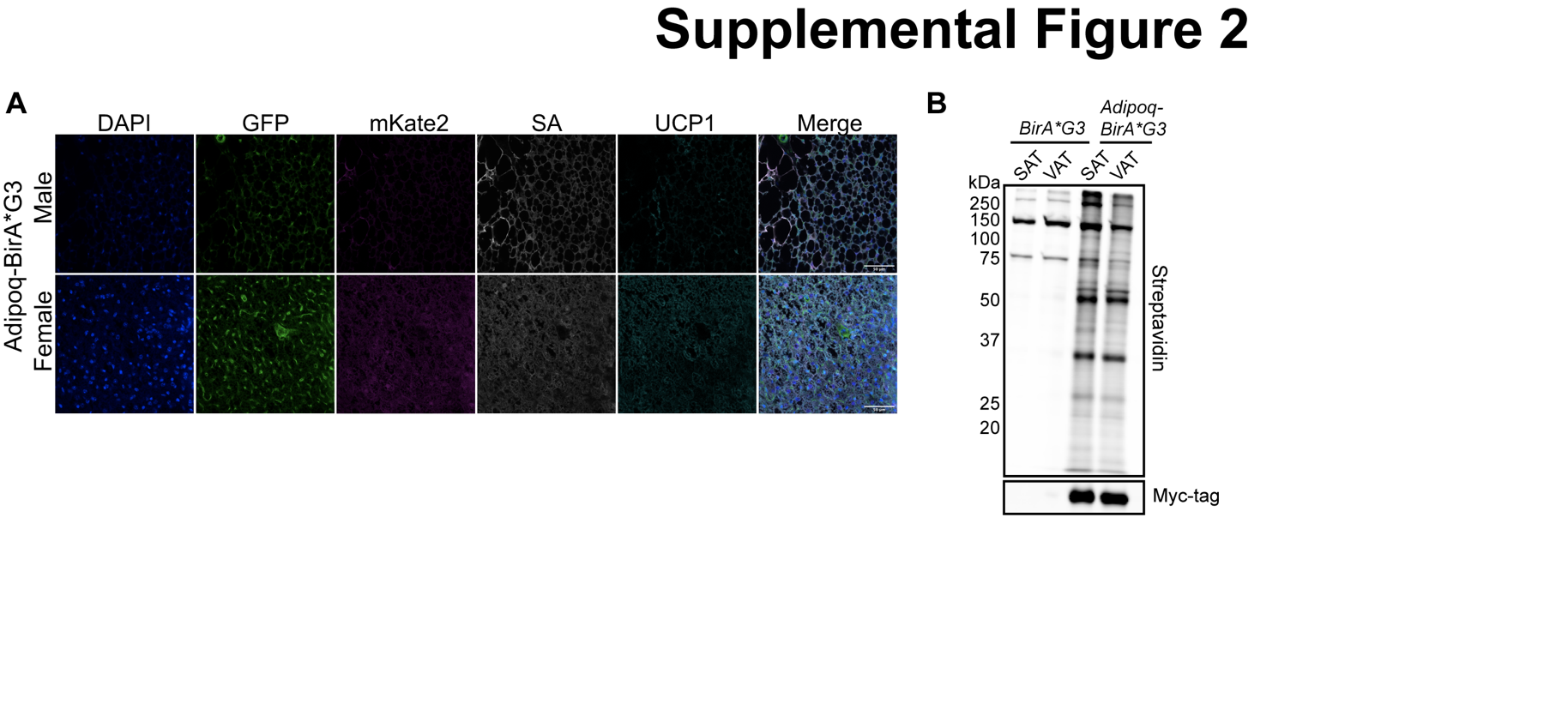
**

***Supplemental Figure 2. Cytoplasmic proximity labeling of brown, subcutaneous, and visceral adipose tissue.***

*(A) Immunofluorescence staining shows expression of native GFP, native mKate2, biotinylated proteins (SA: streptavidin), and UCP1 (BAT marker protein) in the interscapular brown adipose tissue sections from male and female Adipoq-BirA*G3 mice. Scale bar: 50* $\mu m$

*(B) Western blotting of streptavidin affinity purified posterior (SAT) and retroperitoneal (VAT) adipose tissue from Adipoq-BirA*G3 and BirA*G3 (control) mice. Each lane is from individual mice (n=1/genotype). Input: 100* $\mu$*g. Upper: streptavidin, Lower: Myc-tag (~35 kDa).*


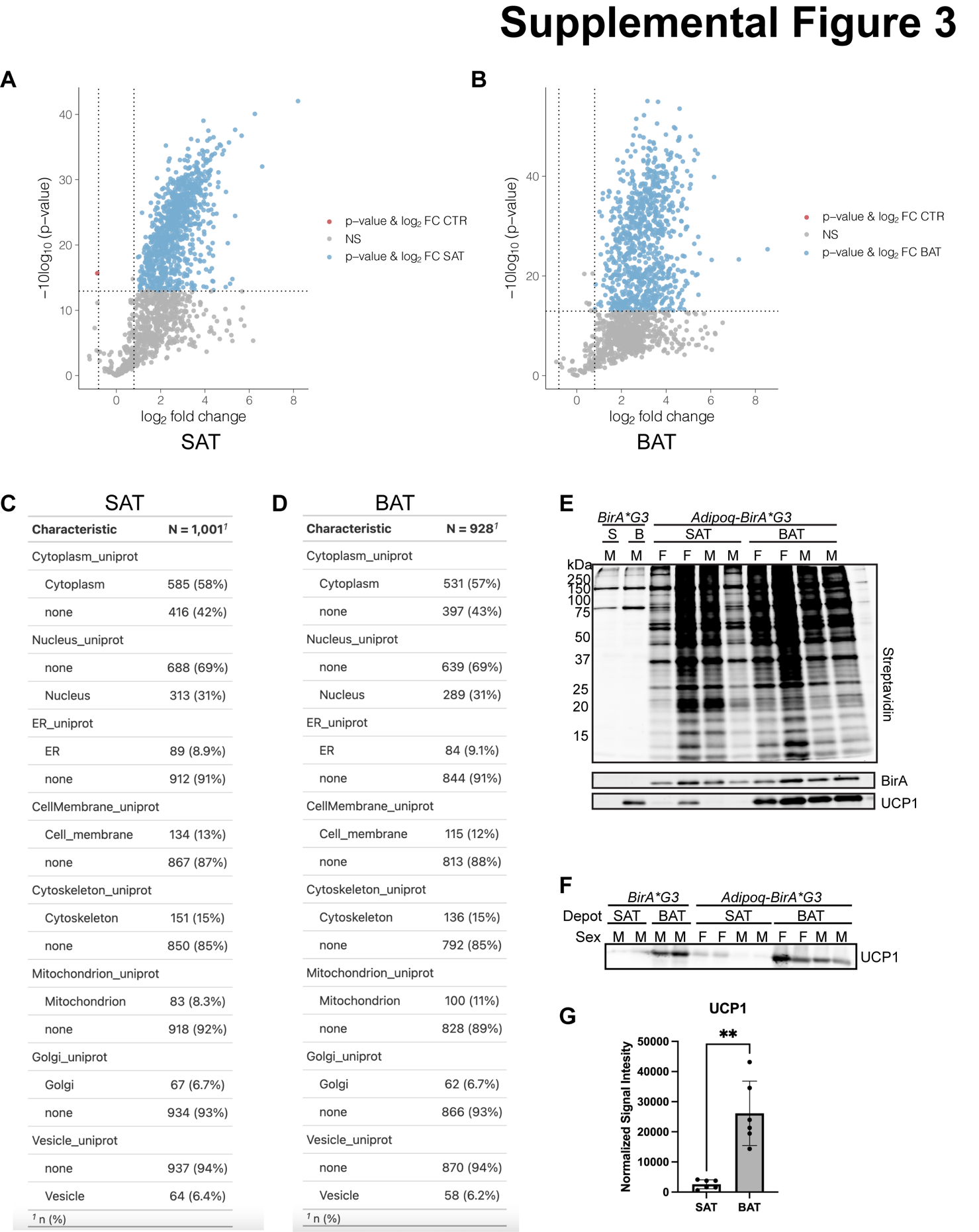


***Supplemental Figure 3. Proteome profiling of male brown and white adipocytes***

*(A) Volcano plot of proteins detected in mice from Adipoq-BirA*G3 (CRE+) and BirA*G3 (CRE-) control samples from subcutaneous white (SAT) depot. Log2FC plotted on x-axis, -10log10(p-value) plotted on y-axis. Significantly enriched proteins (p-value 〈 0.05, log2FC 〉 1.0) shown in blue (CRE+).
(B) Volcano plot of proteins detected in mice from Adipoq-BirA*G3 (CRE+) and BirA*G3 (CRE-) control samples from interscapular (BAT) depot. Log2FC plotted on x-axis, -10log10(p-value) plotted on y-axis. Significantly enriched proteins (p-value 〈 0.05, log2FC 〉 1.0) shown in blue (CRE+)*

*(C) Percent of subcellular localization by Uniprot annotations of enriched proteins (p-value < 0.05, log_2_FC > 1.0) from Adipoq-BirA*G3 subcutaneous adipose tissue over BirA*G3 (control) subcutaneous adipose tissue. Each subcellular compartment annotation was compared individually.*

*(D) Percent of subcellular localization by Uniprot annotations of enriched proteins (p-value < 0.05, log_2_FC > 1.0) from Adipoq-BirA*G3 brown adipose tissue over BirA*G3 (control) brown adipose tissue. Each subcellular compartment annotation was compared individually.*

*(E) Western blot analysis of streptavidin affinity purified proteins from SAT and BAT showing biotinylation levels (streptavidin), BirA*G3 levels, and UCP1 levels in BAT (log_2_FC 3.18 BAT/SAT). Each lane represents one biological sample. F=female, M=male, Upper: streptavidin, Middle: BirA (~35 kDa), Lower: UCP1 (~33 kDa); S=SAT, B=BAT*

*(F) Western blot of total protein lysate (T) from subcutaneous (S, posterior, n=6) and brown (B, interscapular, n=6) adipose tissue in male (M, n=6) and female (F, n=6) Adipoq-BirA*G3 mice and BirA*G3 (control) mice. (n=4/genotype in Cre+ condition) Input: 25 ug lysate. UCP1 (~33 kDa). Total protein stain used for normalized loading in supplemental figure 6A. S=SAT, B=BAT*

*(G) Normalized signal intensity (Supplemental Fig 6A) of UCP1 expression in panel F between SAT (n=6) and BAT (n=6). **=significant; p-value = 0.0014 (Welch’s one-tailed t-test).*


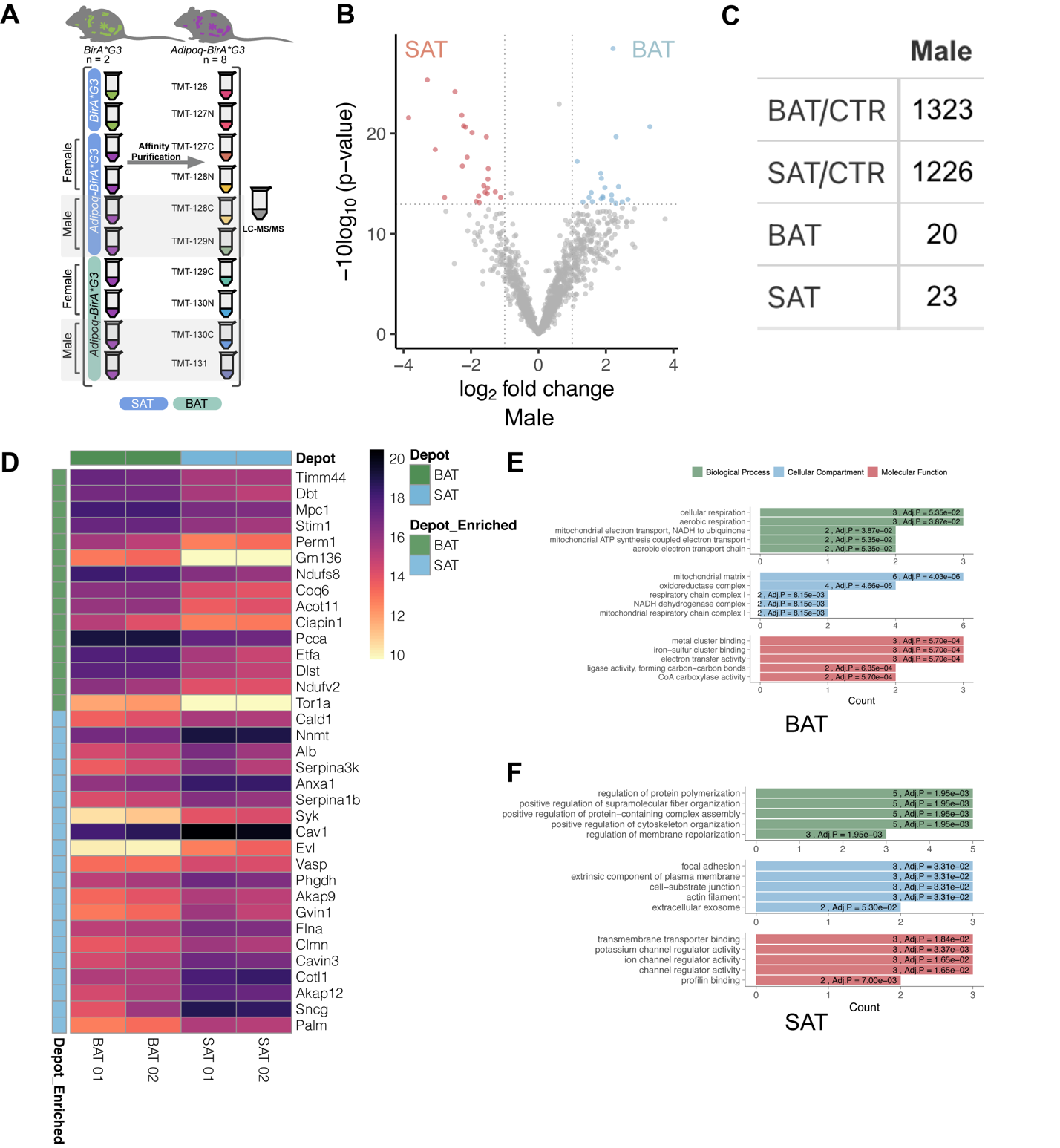


***Supplemental Figure 4. Detection and proteome profiling of male brown and white adipocytes.***

*(A) Tandem Mass Tag (TMT)-based 10plex LC-MS/MS workflow to identify adipocyte proteome differences in males and females between posterior (Pos, white) and interscapular (Int, brown) adipose tissue of Adipoq-BirA*G3 (n=8) and BirA*G3 (n=2) mice.*

*(B) Volcano plot of proteins detected in Adipoq-BirA*G3 male mice from interscapular (Int) BAT and posterior (Pos) SAT depots. Log_2_FC plotted on x-axis, -10log_10_(p-value) plotted on y-axis. Significantly enriched proteins (p-value < 0.05, log_2_FC > 1.0) shown in red (posterior) and blue (interscapular).*

*(C) Summary of significantly enriched proteins (p-value < 0.05, log_2_FC > 1.0) between Adipoq-BirA*G3 and BirA*G3 (CTR) mice and depot specific enriched proteins.*

*(D) Heatmap of depot-specific significantly enriched proteins from panel B.*

*(E) Gene ontology (GO) term analysis for enriched proteins related to biological process (green), cellular compartment (blue), and molecular function (red) for significantly enriched interscapular BAT adipocyte proteins in male Adipoq-BirA*G3 mice.*

*(F) Gene ontology (GO) term analysis for enriched proteins related to biological process (green), cellular compartment (blue), and molecular function (red) for significantly enriched posterior SAT adipocyte proteins in male Adipoq-BirA*G3 mice.*

**
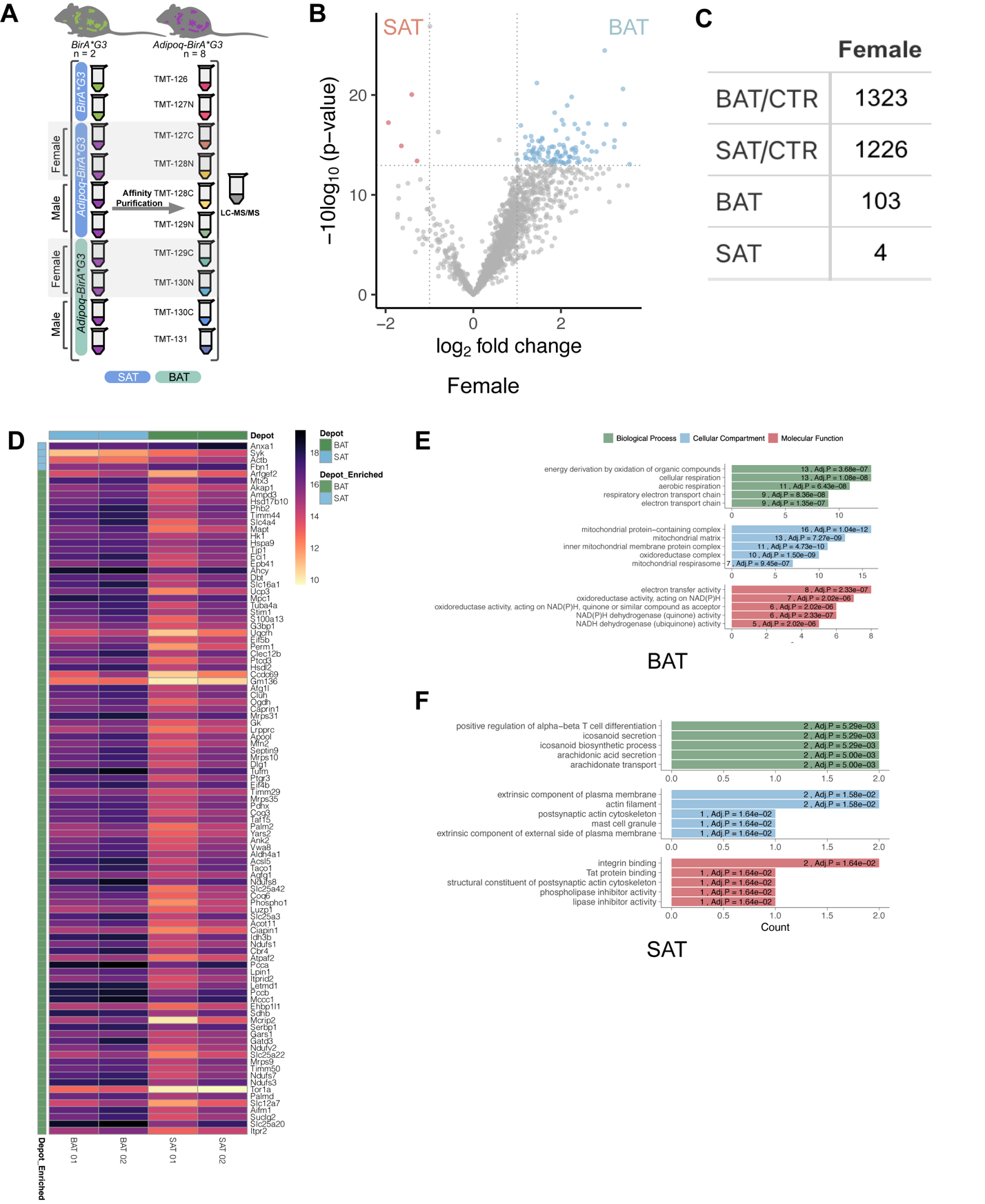
**

***Supplemental Figure 5. Detection and proteome profiling of female brown and white adipocytes.***

*(A) Tandem Mass Tag (TMT)-based 10plex LC-MS/MS workflow to identify adipocyte proteome differences in males and females between posterior (Pos, white) and interscapular (Int, brown) adipose tissue of Adipoq-BirA*G3 (n=8) and BirA*G3 (n=2) mice.*

*(B) Volcano plot of proteins detected in Adipoq-BirA*G3 female mice from interscapular (Int) BAT and posterior (Pos) SAT depots. Log_2_FC plotted on x-axis, -10log_10_(p-value) plotted on y-axis. Significantly enriched proteins (p-value < 0.05, log_2_FC > 1.0) shown in red (posterior) and blue (interscapular).*

*(C) Summary of significantly enriched proteins (p-value < 0.05, log_2_FC > 1.0) between Adipoq-BirA*G3 and BirA*G3 (CTR) mice and depot specific enriched proteins.*

*(D) Heatmap of depot-specific significantly enriched proteins from panel B.*

*(E) Gene ontology (GO) term analysis for enriched proteins related to biological process (green), cellular compartment (blue), and molecular function (red) for significantly enriched interscapular BAT adipocyte proteins in female Adipoq-BirA*G3 mice.*

*(F) Gene ontology (GO) term analysis for enriched proteins related to biological process (green), cellular compartment (blue), and molecular function (red) for significantly enriched posterior SAT adipocyte proteins in female Adipoq-BirA*G3 mice.*

**
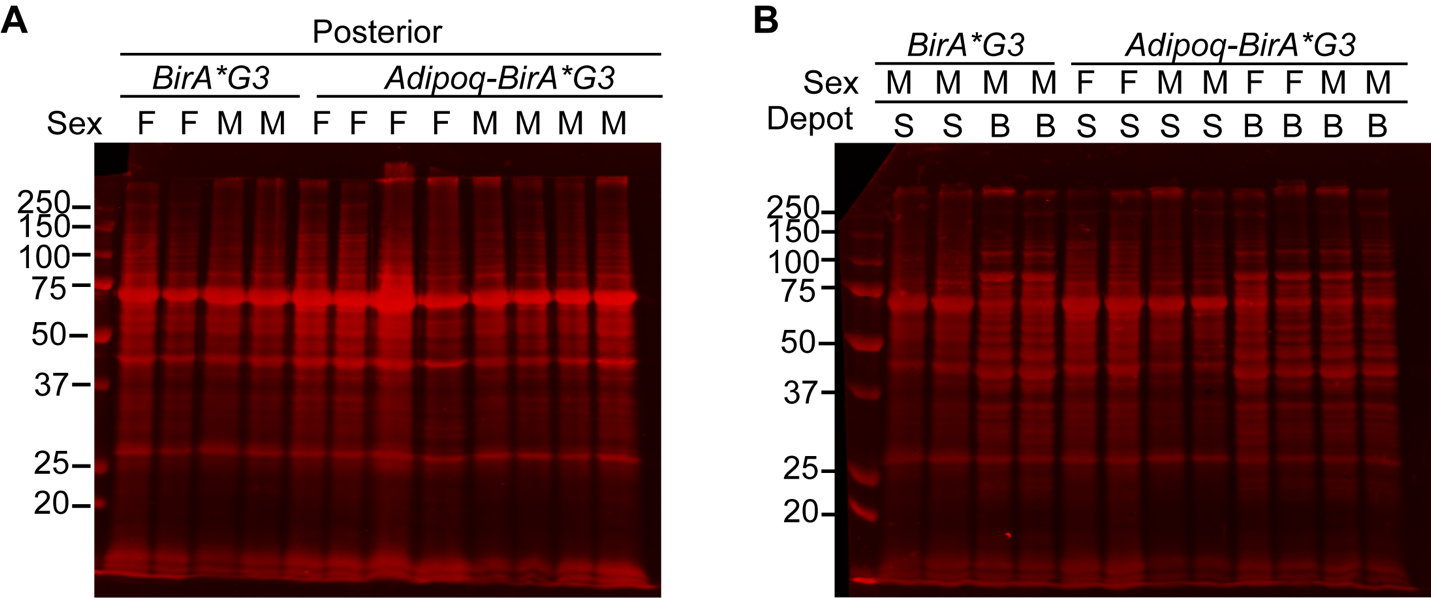
**

***Supplemental Figure 6. Total Protein Stain for Normalization of Western Blots.***

*(A) Total protein stain for signal normalization of western blot (Fig 4D) total protein lysate (T) from subcutaneous posterior adipose tissue in male (M) and female (F) Adipoq-BirA*G3 mice and BirA*G3 (control) mice. (n=4/genotype in Cre+ condition) Input: 25 ug.*

*(B) Total protein stain for signal normalization of western blot (Supplemental Fig 3F, G) total protein lysate (T) from subcutaneous (S, posterior) and brown (B, interscapular) adipose tissue in male (M) and female (F) Adipoq-BirA*G3 mice and BirA*G3 (control) mice. (n=4/genotype in Cre+ condition) Input: 25 ug.*

**
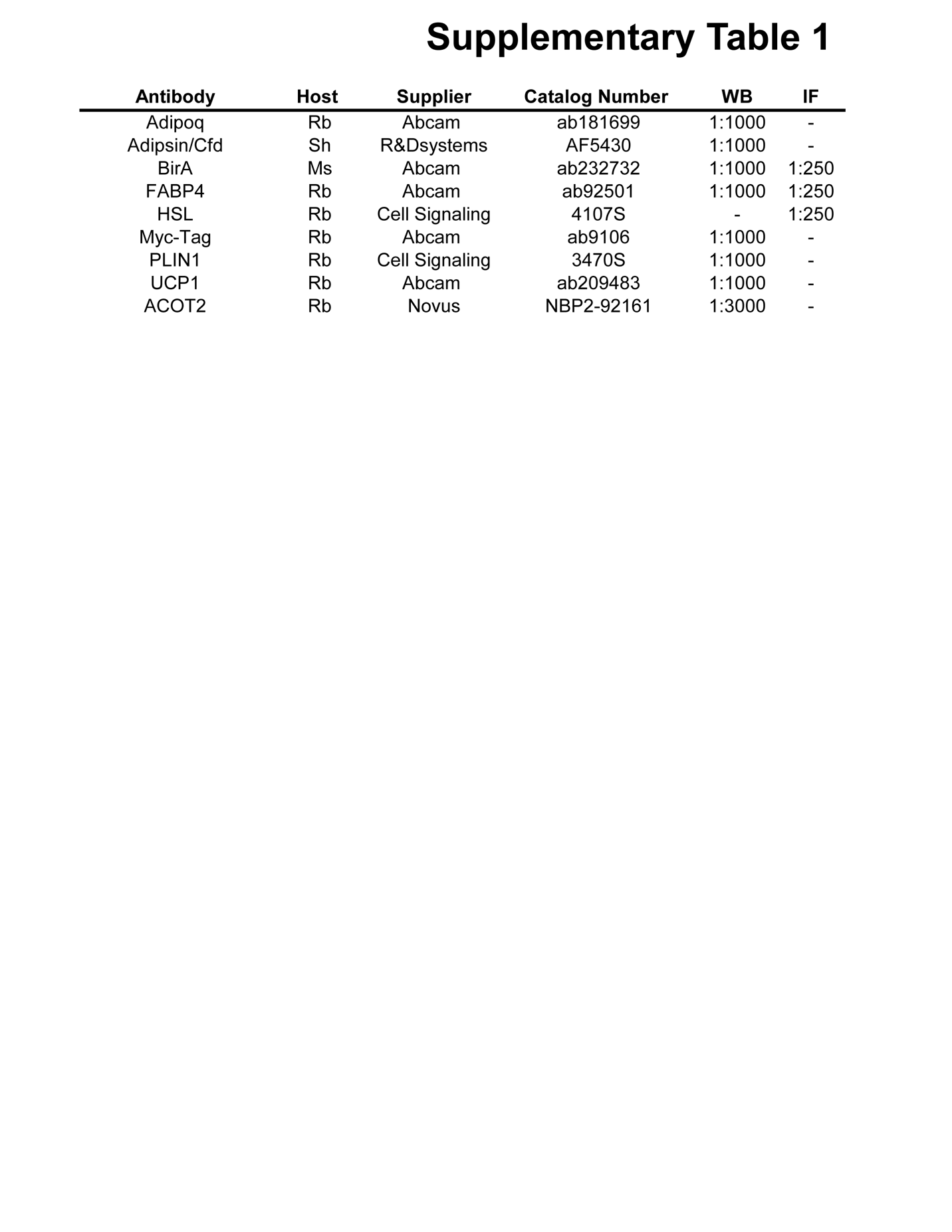
**

***Supplemental Table 1.*** *Primary Antibodies used for Western Blot (WB) and Immunostaining (IF).*

**
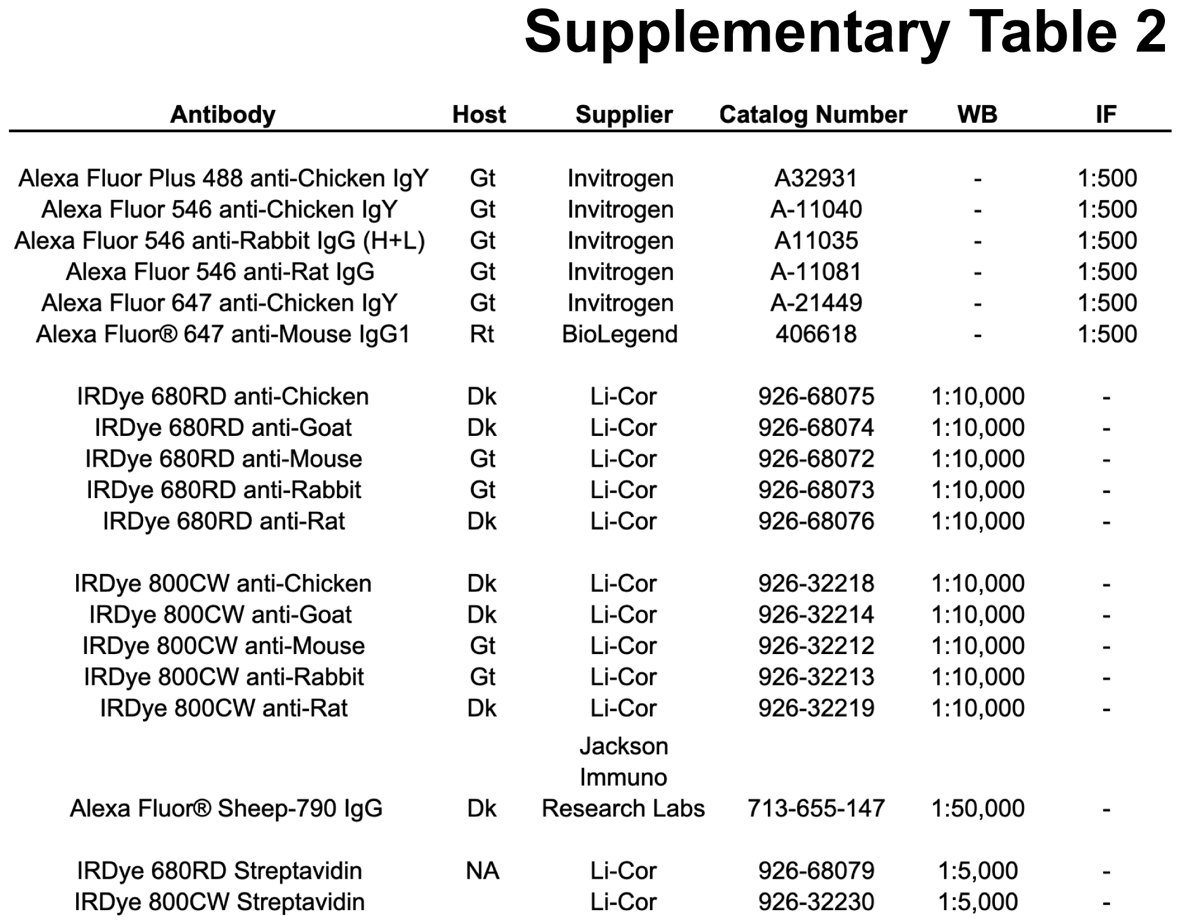
**

***Supplemental Table 2.*** *Secondary Antibodies used for Western Blot (WB) and Immunostaining (IF).*
